# CmABF1 and CmCBF4 cooperatively regulate putrescine synthesis to improve cold tolerance of melon seedlings

**DOI:** 10.1093/hr/uhac002

**Published:** 2022-02-11

**Authors:** Meng Li, Xiaoyu Duan, Ge Gao, Tao Liu, Hongyan Qi

**Affiliations:** 1 College of Horticulture, Shenyang Agricultural University, Shenyang 110866, Liaoning, China; 2 Key Laboratory of Protected Horticulture of Education Ministry and Liaoning Province, Shenyang 110866, Liaoning, China; 3 National and Local Joint Engineering Research Centre of Northern Horticultural, Facilities Design and Application Technology (Liaoning), Shenyang 110866, Liaoning, China

## Abstract

Low temperatures severely restrict melon seedling growth. However, the mechanisms by which melon adapts to cold stress are poorly understood. Arginine decarboxylase (ADC), a key synthetase, catalyzes putrescine biosynthesis in plants. In this study, we found that *CmADC* functions as a positive regulator of melon seedling cold tolerance. In addition, two transcription factors, abscisic acid-responsive element (ABRE)-binding factor 1 (CmABF1) and C-repeat binding factor 4 (CmCBF4), directly target *CmADC* to trigger its expression. Consistently, virus-induced gene silencing (VIGS) of *CmABF1* or *CmCBF4* downregulated *CmADC* abundance, decreased putrescine accumulation, and reduced cold tolerance. Furthermore, some other CBF and ABF members show at least partial functional redundancy and complementarity with CmABF1 and CmCBF4. Overall, our work reveals that the ABA, CBF, and polyamine pathways may form a cooperative regulatory network to participate in plant cold stress response.

## Introduction

Cold stress limits the geographical distribution of thermophilic plants and causes enormous losses in agricultural production. Low temperatures severely hinder plant physiological and biochemical processes, such as photosynthesis, nutrient absorption, and metabolism, resulting in growth stagnation and even death [1]. To survive in cold environments, plants have evolved sophisticated mechanisms to adapt to cold stress [2], one of which is the polyamine pathway [3, 4].

Polyamines (PAs), including putrescine (Put, a diamine), spermidine (Spd, a triamine), and spermine (Spm, a tetramine), possess low molecular weights, polycations, and aliphatic nitrogen-containing substances, and they play important roles in the overall life cycle of plants, from seed germination to fruit ripening, abscission, and senescence [5, 6]. PA biosynthesis begins with the synthesis of Put from L-arginine catalyzed by arginine decarboxylase (ADC), followed by the synthesis of Spd and Spm by spermidine synthase (SPDS) and spermine synthase (SPMS), which requires s-adenosylmethionine (SAM) to provide aminopropyl through decarboxylation [7]. More importantly, there has been a growing interest in the study of PAs involved in plant stresses, including drought, hypoxia, high temperature, low temperature, salinity, and metal toxicity [3, 4, 8, 9]. The main role of PAs in plant stress is to counteract the damage caused by reactive oxygen species (ROS) and to prevent free radical damage or oxidative stress while also modulating ion channels to protect the morphology and integrity of cell membranes, nucleic acids, and proteins [4, 10]. Moreover, PAs interact with hormone pathways (ethylene, jasmonate, auxin, gibberellins, cytokinins, abscisic acid, salicylic acid, and brassinosteroids) or other signaling molecules (Ca^2+^, NO, H_2_O_2_, and gamma-aminobutyric acid) to cope with adverse environments [6, 11–15].

Accumulating evidence suggests that Put synthesis mediated by *ADC* plays a positive role in the stress response of many crops. *ADC* from *Poncirus trifoliata* confers abiotic stress tolerance in *Arabidopsis* for stresses such as osmotic stress, dehydration, drought, and low temperatures [16]. In potato, *SaADC1* is involved in cold-acclimated freezing tolerance [17]. *ADC*-mediated Put biosynthesis is reportedly controlled by various transcription factors (TFs). For example, PbrMYB21 targets *ADC* to modulate polyamine levels and enhance drought tolerance of *Pyrus betulaefolia* [18]. *FcWRKY70* [19] of *Fortunella crassifolia* and *PtrABF* [20] of *P. trifoliata* also function in drought tolerance by promoting Put accumulation through the regulation of *ADC* expression. However, PtrNAC72 negatively regulates *ADC* expression and impairs *P. trifoliata* drought tolerance [21]. As the specific receptor or signal transduction mechanism of PAs has not been explored, the mechanisms by which PAs regulate plant stress resistance have yet to be fully understood [6]. To date, *ADC* is only reported to be induced by salt stress in cucurbit seedlings, such as cucumber [22] and muskmelon [23, 24]. However, the complex regulatory module of *ADC*-mediated Put biosynthesis in cucurbit crops has not been fully elucidated.

During the past two decades, an emerging trend of crosstalk between the phytohormone abscisic acid (ABA) and PAs in response to ambient pressure has been observed [4, 6, 12, 25, 26]. ABA is also widely involved in plant growth and development, as well as in a variety of biotic and abiotic stresses [27]. Moreover, ABA biosynthesis is catalyzed by a series of enzymes with β-carotene as a precursor through the carotenoid pathway. Among them, 9-cis-epoxy carotenoid dioxygenase (NCED) is the rate-limiting enzyme in this process [28–30]. The core components of ABA signaling are a dual inhibitory system composed of pyrabactin resistance/pyrabactin resistance-like/regulatory component of ABA receptor (PYR/PYL/RCAR), type 2C protein phosphatase (PP2C), SNFl-related protein kinase 2 (SnRK2), and abscisic acid-responsive element (ABRE)-binding factors (ABFs) [27, 31, 32]. Reciprocal complementation tests have shown that Put and ABA regulate each other’s synthesis through positive feedback in response to stress [12]. Transcript levels of *ADCs*, *SPDS*, and *SPMS* were impaired in various ABA-deficient *Arabidopsis* mutants [30, 33]. ABA treatment can induce PA accumulation in many plants, such as *Arabidopsis* [34], wheat [35], ice plant [36] and grapevine [37, 38]. Notably, the above reports all consistently mentioned that ABA treatment stimulated Put accumulation, but its effects on Spd and Spm varied [34–38]. Put promotes ABA accumulation by inducing *NCED* transcription and inhibiting ABA degradation under cold stress in *Arabidopsis* [25], drought stress in *Lotus tenuis* [39], and cadmium stress in *Scrophularia striata* [40]. In addition to the ABA pathway, DREB1/CBF (dehydration responsive element binding protein1/C-repeat binding factor) is another crucial and well-documented signaling pathway in the cold regulatory network [2, 41].

CBFs occupy the crossroads of the transcriptional regulatory network that underlies cold stress [41, 42]. They can directly bind to DRE/CRT (dehydration-responsive element/C repeat) *cis*-acting elements in the promoters of *COR* (cold regulated) genes and trigger their expression to withstand the bitter cold, and approximately 10%–20% of *COR* genes are regulated by three CBFs in *Arabidopsis* [42, 43]. In *Arabidopsis* or rice, the transcription of *CBF* genes is positively or negatively controlled by various TFs, hormone signaling components, protein kinases and phosphatases, epigenetic regulation, and post-translational regulation [1, 2, 41, 42, 44, 45]. Such regulating factors include ICE1/2 (inducer of CBF expression 1/2), MYB15, CAMTAs (calmodulin-binding transcription activators), PIF3/4/7 (phytochrome-interacting factor 3/4/7), EIN3 (ethylene insensitive 3), JAZ1/4 (jasmonate zim-domain protein 1/4), BZR1 (brassinazole resistant 1), BES1 (BRI1-EMS-SUPPRESSOR1), miR397, lnRNA, OST1 (open stomata 1), MPK3/6 (MAP KINASE3/6) and histone acetylation by GCN5 (general control non-derepressible 5), and HOS1 (high expression of osmotically responsive gene 1).

A novel view is emerging that ABA- and CBF-independent pathways do not respond to low temperatures independently [44]. OST1 and MYB96 are an ABA-induced kinase and TF, respectively, which crosstalk with the CBF pathway via ICE1/2 or CAMTA [46, 47]. Furthermore, some *COR* genes such as *COR15A*, *COR47*, *RD29A*, and *RD22* contain both DRE (TACCGACAT) and ABRE (ABA-responsive element, ACGTGG/TC) in their promoters [48]. Notably, there are 2052 genes in *Arabidopsis* harboring the two *cis*-elements [49], indicating that they may be jointly regulated by the ABF and CBF subfamilies in response to stress.

Melon, which originated in tropical and sub-tropical areas but is now widely cultivated worldwide, is vulnerable to cold damage in temperate latitudes [50, 51]. Evidence suggests that exogenous ABA can enhance cold tolerance of oriental melon seedlings [52], and PA accumulation contributes to adaptation to root-zone hypoxia stress [53], Ca(NO_3_)_2_ stress [23], and salinity-alkalinity stress in melon seedlings [24], as well as cold stress in melon fruit [54]. Only two *CmCBFs*, *CmCBF1* and *CmCBF3*, have been identified, and their expression was positively correlated with melon fruit cold tolerance [54]. Whether the PA, ABA, and CBF pathways synergistically regulate melon cold tolerance remains largely unknown.

In a preliminary experiment, we found that among the three PAs (Put, Spd, and Spm), only Put was present at significantly higher levels in a cold-tolerant genotype than in a cold-sensitive genotype. In this study, we first found that cold treatment caused *CmADC* upregulation and Put accumulation in melon seedlings. Sequence analysis showed that the *CmADC* promoter harbored at least three ABRE and three DRE motifs. Second, we isolated four *CmCBFs* and five *CmABFs*, which were significantly induced in response to cold stress. Among them, CmABF1 and CmCBF4 were selected as candidate TFs that could directly bind to promoter fragments of *CmADC in vitro* and *in planta* to promote its transcription. Virus-induced gene silencing (VIGS) assays further showed that *CmABF1* and *CmCBF4* played positive roles in the cold tolerance of melon seedlings by promoting Put synthesis. This study provides new evidence that the ABA and CBF pathways in cold response are not entirely independent and that *CmADC* is at the junction of these pathways.

## Results

### *CmADC*-mediated Put accumulation enhances cold tolerance of melon seedlings

**Figure 1:**
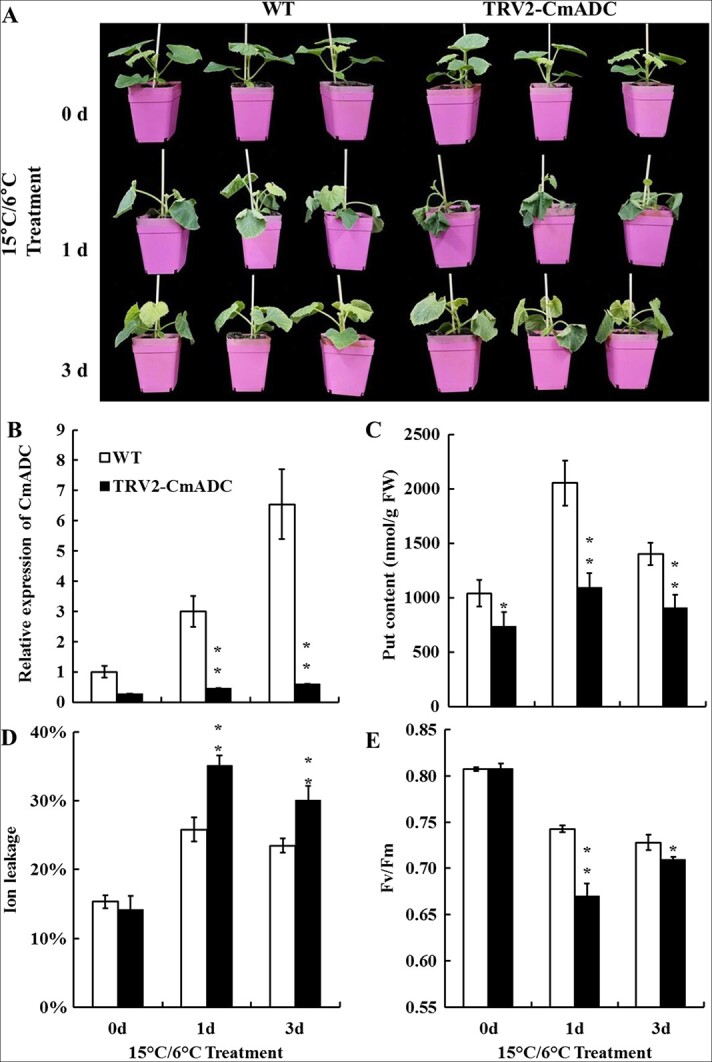
**Silencing of *CmADC* in melon seedlings by virus-induced gene silencing (VIGS) resulted in elevated cold sensitivity.** Plant phenotype (**A**), *CmADC* expression (**B**), Put accumulation (**C**), ion leakage (**D**), and Fv/Fm (**E**) of TRV2-empty (WT) plants and *CmADC*-silenced plants under low-temperature treatment (15°C/6°C). An independent *t*-test was used to analyze the difference between the treatment and control groups. Significant differences are marked with asterisks (**P < 0.01; *P < 0.05). Error bars are shown with three biological replicates.

Only one arginine decarboxylase (ADC) gene was identified in the melon genome (GenBank No. MZ416921). To examine whether Put functions in response to cold stress, *CmADC* was silenced by the VIGS system (Fig. S1). First, we found that low temperature could induce *CmADC* expression (Fig. 1B) and Put accumulation in wild-type (WT) plants (Fig. 1C), suggesting that Put positively participates in the response of melon seedlings to low temperature. Compared with the WT plants, the silenced plants wilted more seriously after a 1-day treatment, and irreversible damage occurred after a 3-day treatment (Fig. 1A). *CmADC* expression and Put levels decreased sharply in silenced plants (*P* < 0.01) (Fig. 1B–C). Moreover, the cell membrane was seriously damaged, as ion leakage markedly increased (*P* < 0.01) (Fig. 1D) and the maximum photochemical efficiency of photosystem II (Fv/Fm) significantly decreased (*P* < 0.05) (Fig. 1E), indicating that the blockage of Put synthesis caused melon seedlings to be more sensitive to low temperatures. Notably, exogenous Put supplementation could proportionately compensate for the decrease in cold tolerance caused by *CmADC* silencing (Fig. S2). These results demonstrated that *CmADC*-mediated Put accumulation functions in the cold tolerance of melon seedlings.

### Cloning and activity analysis of the *CmADC* promoter

**Figure 2:**
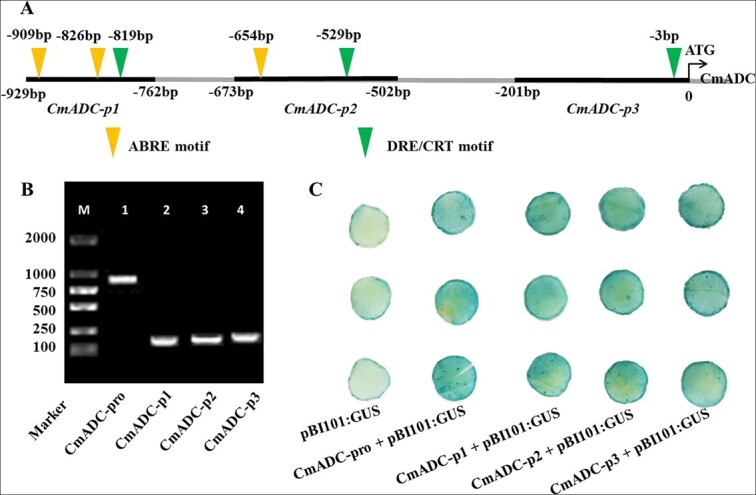
**Cloning and activity analysis of the *CmADC* promoter. A** Diagram of the 929-bp *CmADC* promoter, including the locations of the *CmADC-p1*, *CmADC-p2*, and *CmADC-p3* fragments and the ABRE and DRE/CRT motifs. **B** The lengths of the four cloned *CmADC* promoter fragments (*CmADC-pro*, *CmADC-p1*, *CmADC-p2*, and *CmADC-p3*) were 929 bp, 168 bp, 172 bp, and 201 bp, respectively. **C** Four *CmADC* promoter fragments were inserted upstream of the mini35S promoter to initiate the GUS gene. The GUS gene was differentially expressed in tobacco leaves transformed with the indicated vectors. The staining level represents the abundance of GUS protein in the leaves.

To explore the regulatory factors of *CmADC*, we cloned a 929-bp fragment of its promoter region, which we named *CmADC-pro* (Fig. 2A, B). *CmADC-pro* contained three DREB motifs and three AREB motifs (Fig. 2A). Therefore, we speculated that CmABF and CmCBF might regulate *CmADC* expression. Based on the location of the two *cis*-elements, three truncated fragments were cloned and named *CmADC-p1* (168 bp), *CmADC-p2* (172 bp), and *CmADC-p3* (201bp), respectively (Fig. 2A, B). All four fragments showed promoter activity (Fig. 2C).

### Gene identification of *CmCBF* and *CmABF*

**Figure 3:**
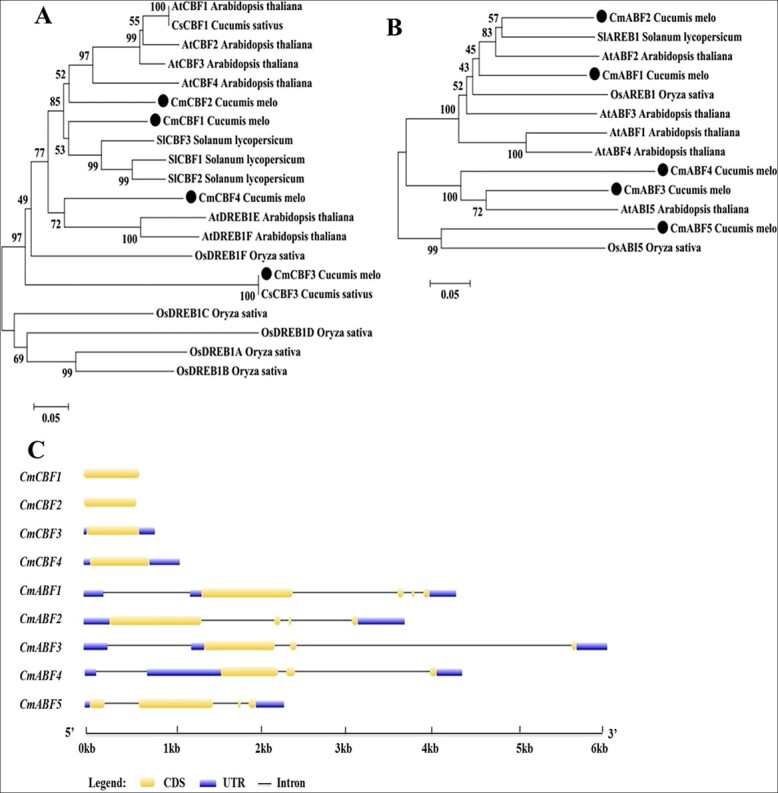
**Phylogenetic analysis and gene structures of CmCBF and CmABF.** **A** and **B** An NJ phylogenetic tree was constructed using a model monocotyledon (*Oryza sativa*) and dicotyledons (*Arabidopsis thaliana*, *Solanum lycopersicum*, and *Cucumis melo*). An *ABF* has not been reported in cucumber, and it has therefore not been included in the phylogenetic tree. **C** Gene Structure Display Server 2.0 software (http://gsds.gao-lab.org) was used to investigate the exon-intron organization of these genes.

Previous studies have reported two *CmCBFs* in melon: *CmCBF1* (GenBank No*.* AMK37721) and *CmCBF3* (GenBank No*.* AMK37722) [54]. We identified two new *CmCBFs*, *MELO3C005367* and *MELO3C005629*, named *CmCBF2* (GenBank No*.*MZ402513) and *CmCBF4* (GenBank No*.*MZ402514), respectively (Table S9). The four *CmCBFs* were randomly distributed on three chromosomes and were distributed unevenly across the clades. *CmCBF2* and *CmCBF4* were orthologous to *AtCBFs*, whereas *CmCBF1* was orthologous to *SlCBFs*, and *CmCBF3* had a close phylogenetic relationship with *CsCBF3* (Fig. 3A). There were no introns in the four *CmCBF* genes (Fig. 3C).

We identified five *CmABFs* distributed on four different chromosomes (Table S9). *CmABF1* (GenBank No*.*MZ389323) and *CmABF2* (GenBank No*.*MZ402509) were in the same group, which included *ABF* members from monocotyledons (*Oryza sativa*) and dicotyledons (*Arabidopsis thaliana*, *Solanum lycopersicum*) with highly divergent and relatively low bootstrap values (Fig. 3B). *CmABF3* (GenBank No*.*MZ402510) and *CmABF4* (GenBank No*.*MZ402511) were clustered with *AtABI5* (abscisic acid-insensitive 5), and *CmABF5* (GenBank No*.*MZ402512) was grouped with *OsABI5* (Fig. 3B). The *CmABFs* contained three or four exons and exhibited different intron/exon arrangements (Fig. 3C).

### Gene expression, subcellular localization, and transcriptional activity of CmCBFs and CmABFs

**Figure 4:**
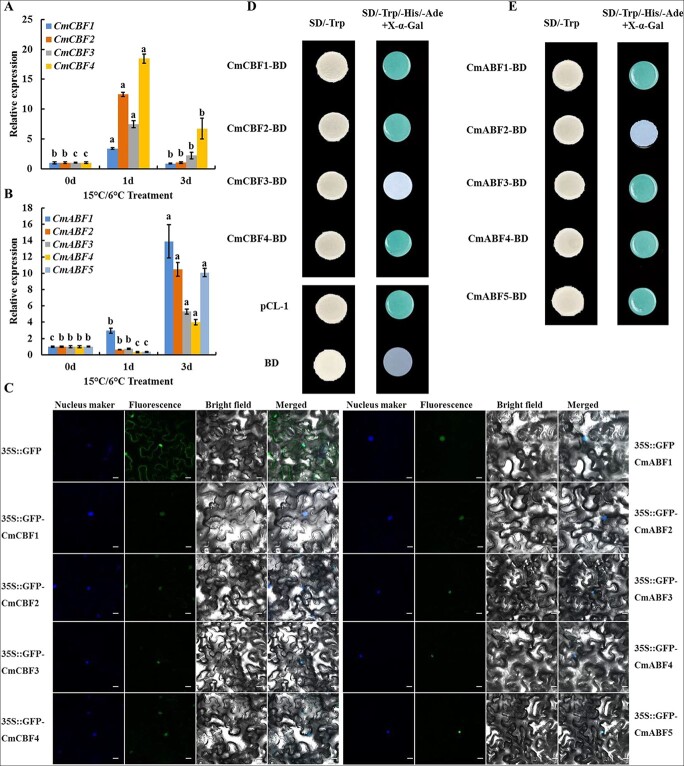
**Expression, subcellular localization, and transcriptional activity of CmCBFs and CmABFs. A** and **B** Relative expression levels of *CmCBFs* and *CmABFs* in melon leaves exposed to 15°C/6°C for 0, 1, and 3 days. Values are means of three biological replicates. Bars indicate SD. Significant differences are indicated at the level of *P* < 0.05 with lowercase letters based on Duncan’s multiple range test. **C** Subcellular localization of CmCBFs and CmABFs. *35S::GFP-CmCBF1(2/3/4)* and *35S::GFP-CmABF1(2/3/4/5)* were separately transiently expressed in tobacco leaves and visualized by confocal microscopy (×40). The nucleus was dyed with 4,6-diamidino-2-phenylindole (DAPI). **D** and **E** Transcriptional activation of CmCBFs and CmABFs in yeast cells. Y2H Gold strains expressing pCL-1, *pGBKT7* empty vector (BD), and *pBD-CmCBF1(2/3/4)* or *pBD-CmABF1(2/3/4/5)* were cultured on SD/−Trp or selective SD/−Trp/−His/−Ade medium. *pGBKT7* empty vector and *pCL-1* were used as the negative and positive controls, respectively

To verify whether CmABF and CmCBF were candidate TFs that regulated *CmADC* expression, we first detected *CmCBFs* or *CmABFs* expression at low temperatures (15°C/6°C). *CmCBF* genes were induced rapidly by cold treatment (*P* < 0.05), and the expression peak of *CmCBF* genes appeared after 1 day of cold induction, especially for *CmCBF4* (Fig. 4A). All five *CmABFs* were induced by cold treatment (*P* < 0.05), especially after a 3-day treatment (Fig. 4B). Only *CmABF1* was upregulated on the first day.

To examine the subcellular localization of the four CmCBFs and five CmABFs, four *35S::CmCBF-GFP* and five *35S::CmABF-GFP* constructs were separately infiltrated into *Nicotiana benthamiana* leaves via *Agrobacterium*-mediated transient transformation. GFP fluorescence was detected only in the nuclei of tobacco cells infiltrated with one of the nine fusion proteins, whereas GFP fluorescence was evenly distributed throughout the tobacco cells that were infiltrated with the *pCAMBIA1300-GFP* empty vector (Fig. 4C). Each of the four CmCBFs and five CmABFs was localized to the nucleus *in vivo*.

The transcriptional activation activities of four CmCBFs and five CmABFs were evaluated in a yeast system. Y2H Gold yeast strains transformed with *pBD-CmCBF1*, *pBD-CmCBF2*, *pBD-CmCBF4*, *pBD-CmABF1*, *pBD-CmABF3*, *pBD-CmABF4*, *pBD-CmABF5*, and the positive control construct (*pCL-1*) grew normally on SD/−Trp/−His/−Ade selective medium and displayed α-galactosidase activity, whereas yeast strains carrying the negative control constructs *pGBKT7*, *CmCBF3*, and *CmABF2* did not grow on the selective medium (Fig. 4D–E). These results indicated that CmCBF1/2/4 and CmABF1/3/4/5 were transcriptional activators, whereas CmCBF3 and CmABF2 were not.

*CmCBF4* and *CmABF1* expression levels were the most pronounced and the earliest to be induced; they were also consistent with *CmADC* expression (Fig. 1B). Therefore, these two genes were selected as the predominant TFs regulating *CmADC* expression.

### CmABF1 and CmCBF4 act as transcriptional activators of *CmADC*

**Figure 5:**
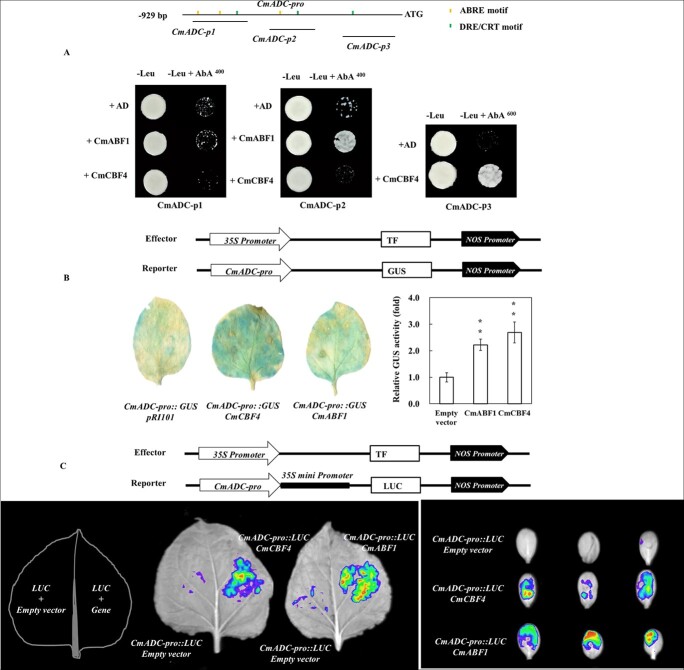
**CmABF1 and CmCBF4 bind directly to the promoter of *CmADC* and act as transcriptional activators. A** Y1H analysis of CmABF1 and CmCBF4 binding to *CmADC* promoter fragments (*CmADC-p1*, *CmADC-p2*, and *CmADC-p3*). The growth status of transformed yeast on two media is shown. Normal yeast growth on defective medium containing the antibiotic aureobasidin A indicates that CmABF1 and CmCBF4 can bind to the promoter of *CmADC*. **B** GUS activity assays of CmABF1 and CmCBF4 binding to the *CmADC* promoter. The histochemical analysis of *GUS* gene activity in tobacco leaves is shown. The staining level represents the abundance of GUS protein in the leaves. Increased relative GUS activity indicates that transcriptional regulation is activated. An independent *t*-test was used to analyze the difference between the treatment and control groups. Significant differences are marked with asterisks (^**^P < 0.01; ^*^P < 0.05). Error bars are shown with three biological replicates. **C** Luciferase reporter assay showing the *in vivo* binding of CmABF1 and CmCBF4 to the *CmADC* promoter. The infected tobacco and melon cotyledons were measured with an *in vivo* fluorescence imager. Fluorescence intensity stronger than that of the control (empty vector) indicates that the TF interacts with the promoter and activates gene expression.

To verify that CmABF1 and CmCBF4 can interact with *CmADC*, Y1H assays were performed *in vitro*. Promoter structure analysis revealed that multiple *cis*-regulatory elements were predicted by PlantCARE. The promoter segment baits (*CmADC-p1*, *CmADC-p2*, and *CmADC-p3*) were fused to the prey vectors, and *pGADT7-CmCBF4* or *pGADT7-CmABF1* was introduced into the Y1H Gold yeast strains. The results suggested that CmABF1 and CmCBF4 could directly bind to the *CmADC-p2* and *CmADC-p3* fragments, respectively (Fig. 5A).

We then investigated how CmABF1 and CmCBF4 interact with the *CmADC* promoter using GUS activation assays (Fig. 5B). The coding sequences (CDSs) of *CmABF1* or *CmCBF4* were singly inserted into a *pRI101* vector as effectors. The *CmADC-pro* promoter fragments were introduced into the *pBI101* vector as reporters. When *35S::CmCBF4* or *35S::CmABF1* was co-infiltrated with *CmADC-pro::GUS*, the GUS protein abundance and the GUS activity level in tobacco leaves were clearly increased (*P* < 0.01) (Fig. 5B), implying that CmCBF4 and CmABF1 function as transcriptional activators. To further confirm this result, luciferase reporter assays were performed in tobacco and melon; *CmADC-pro* was fused to the *pRI-mini35S-LUC* vector and co-expressed with *35S::CmCBF4* or *35S::CmABF1* in tobacco and melon. Surprisingly, the fluorescence signal was higher in the presence of both the effector and reporter constructs than in the control in both tobacco and melon cotyledons (Fig. 5C). These results suggest that CmABF1 and CmCBF4 act as transcriptional activators of *CmADC*.

In addition, we demonstrated that CmCBF1, CmCBF2, CmABF3, CmABF4 and CmABF5 can also directly bind to the *CmADC* promoter and positively regulate its transcription (Fig. S3).

### CmABF1 and CmCBF4 regulate *CmADC* to promote Put accumulation in response to low temperature

**Figure 6:**
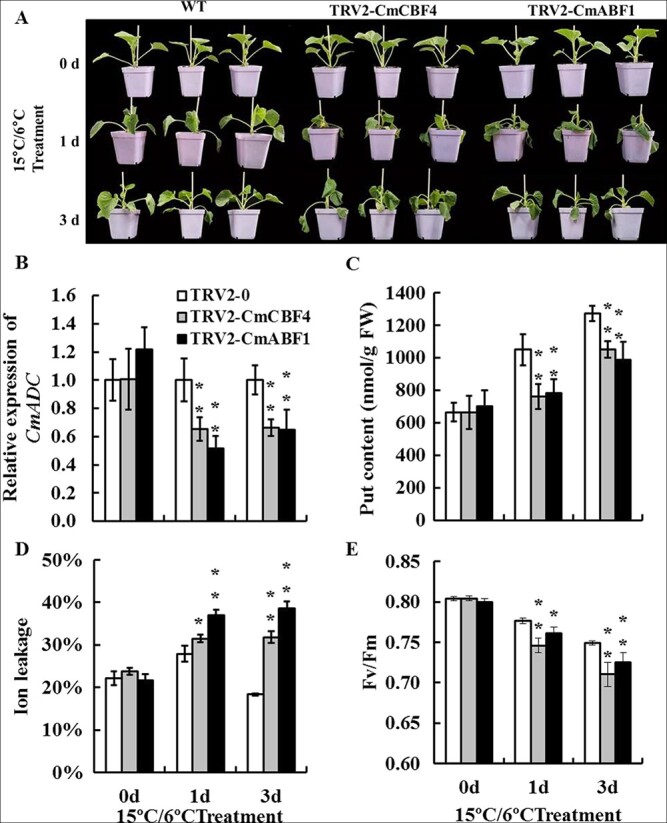
**VIGS of *CmABF1* and *CmCBF4* resulted in a decrease in Put content and cold tolerance of melon seedlings.** Plant phenotype (**A**), *CmADC* expression (**B**), Put accumulation (**C**), ion leakage (**D**), and Fv/Fm (**E**) of *CmABF1-* and *CmCBF4*-silenced seedlings under cold treatment (15°C/6°C). An independent *t*-test was used to analyze the difference between silenced plants and control plants (WT). Significant differences are marked with asterisks (^**^P < 0.01; ^*^P < 0.05). Error bars are shown with three biological replicates.

To further elucidate the role of *CmABF1* and *CmCBF4* in cold tolerance, gene-silenced lines of *TRV2-CmCBF4* and *TRV2-CmABF1* were obtained using the VIGS system to suppress their expression (Fig. S1). Upon exposure to cold, *TRV2-CmCBF4* and *TRV2-CmABF1* plants displayed more severe wilting than WT plants (*P* < 0.01) (Fig. 6A). *CmADC* expression in *CmABF1-* and *CmCBF4*-silenced plants was consistently lower than that in WT plants after 1-day and 3-day cold treatments (*P* < 0.01) (Fig. 6B), and this was accompanied by a decrease in Put content (*P* < 0.01) (Fig. 6C). The ion leakage of both silenced lines was markedly higher than that of WT plants during cold treatment (*P* < 0.01) (Fig. 6D). By contrast, the Fv/Fm values gradually decreased, especially on the third day (*P* < 0.01) (Fig. 6E). Silenced plants treated with exogenous Put suffered less injury; at least in part, the plant phenotype could be recovered to the WT level (Fig. S2). These results indicated that VIGS of *CmCBF4* or *CmABF1* in melon seedlings notably blocked Put biosynthesis, and this was accompanied by compromised cold tolerance.

In addition, VIGS lines of *CmCBF1*, *CmCBF2*, *CmABF3*, *CmABF4* and *CmABF5* showed similar characteristics (Fig. S4), suggesting that they may have functional redundancy and complementarity with CmABF1 and CmCBF4.

## Discussion

PAs, ABA, and CBF are important participants in plant stress responses, and their roles at low temperatures are gradually becoming clearer [2, 4, 27]. In this study, we first tested whether PAs, ABA, and CBF are involved in the cold tolerance of melon seedlings. For PAs, Put accumulated significantly upon cold stress in WT plants and decreased in *CmADC*-silenced plants (Fig. 1), consistent with reports of *ADC* gene upregulation and Put accumulation under cold stress in other thermophilic plants, such as tomato and potato [14, 17]. For ABA pathways, *CmNCED3*-silenced seedlings had higher sensitivity to low temperature and lower cold tolerance (Fig. S5). Meanwhile, as the terminal TFs of the ABA signaling pathway, *CmABFs* were also triggered by low temperature (Fig. 4B), and single-gene silencing compromised the cold tolerance of melon seedlings (Fig. 6). These results strongly suggest that the ABA pathway is involved in the cold tolerance of melon seedlings and may regulate downstream genes in response to low temperature through ABFs [55]. For the CBF pathway, a previous study reported that *CmCBF1* and *CmCBF3* are upregulated in melon fruits during low-temperature storage [54]. Our results showed that all four *CmCBFs* are induced by low temperatures (Fig. 4A). Meanwhile, single-gene silencing of *CmCBFs* or *CmICE1* seriously impaired the cold tolerance of melon seedlings (Fig. 6, S6). Therefore, our results convincingly demonstrate that the PA, ABA, and CBF pathways contribute to melon seedling cold tolerance.

Furthermore, as plant defenses against low temperatures are controlled by sophisticated regulatory networks, we explored whether there is a series–parallel connection among the three pathways.

*ADC*-mediated Put biosynthesis plays an important role in various abiotic stresses [4]. Previous studies have reported that several TFs, such as PbrMYB21 [18], FcWRKY70 [19], PtrABF [20], and PtrNAC72 [21], are involved in the regulatory process. These TFs were demonstrated to interact with elements in the promoter of the corresponding ADC gene (e.g. MYB-recognition motif, CACG-motif, W-box, and ABRE) [18–21]. ABF and CBF can each bind directly to the ABRE and DRE motifs of target genes [2, 49]. Intriguingly, we found that the *CmADC* promoter contains each of the two types of motif (Fig. 2), and subsequent tests have shown that CmABF1 and CmCBF4 can bind to the *CmADC* promoter and promote its transcription in melon. Furthermore, *CmADC* expression and Put accumulation decreased in single-gene-silenced *CmABF1* or *CmCBF4* plants, and this was accompanied by impaired plant cold tolerance (Fig. 6), indicating that these TFs mediated Put biosynthesis to enhance the cold tolerance of melon seedlings by triggering *CmADC* expression. These results demonstrated that CmABF1 and CmCBF4 were similar to PbrMYB21 [18], FcWRKY70 [19], and PtrABF [20], which also play positive roles in abiotic stress.

We found that CmABF1 and CmCBF4 are not the only members that regulate *CmADC* expression; the other family members that performed similar functions included CmCBF1, CmCBF2, CmABF3, CmABF4, and CmABF5 (Figs. S3, S4). Notably, the seven TFs (CmABF1/3/4/5 and CmCBF1/2/4) did not function during the entire process of cold treatment (Fig. 6, S4), indicating that they may have redundant and complementary roles in the regulation of cold tolerance in melon seedlings. Conversely, *CmADC* expression and Put levels did not decrease in some silenced lines, but their cold tolerance was still decreased, suggesting that these TFs may partly respond to low temperature by regulating other target genes, such as *CORs* and *RDs* [42, 59]; identification of the specific target genes requires further research.

Both ABF and CBF are terminal TFs in signal transduction pathways, and they are also regulated by a variety of TFs or kinases [27, 42], such as ICE and SnRK2. After silencing *CmICE1*, melon seedlings were more sensitive to low temperatures, and both *CmADC* abundance and Put content decreased sharply (Fig. S6). In *Arabidopsis*, ICE1 specifically recognizes the MYC motif in the *CBF3* promoter [60]. The MYC motif also exists in the *CmADC* promoter, suggesting that CmICE1 might also directly regulate *CmADC* in melon seedlings under low temperature. However, the detailed mechanisms have not yet been investigated. In addition, SnRK2 works upstream of ABF, activating it [55]. To the best of our knowledge, SnRKs and ABA receptors have not been identified in the melon genome, and the complex regulatory mechanism of this pathway requires further exploration.

## Conclusion

**Figure 7:**
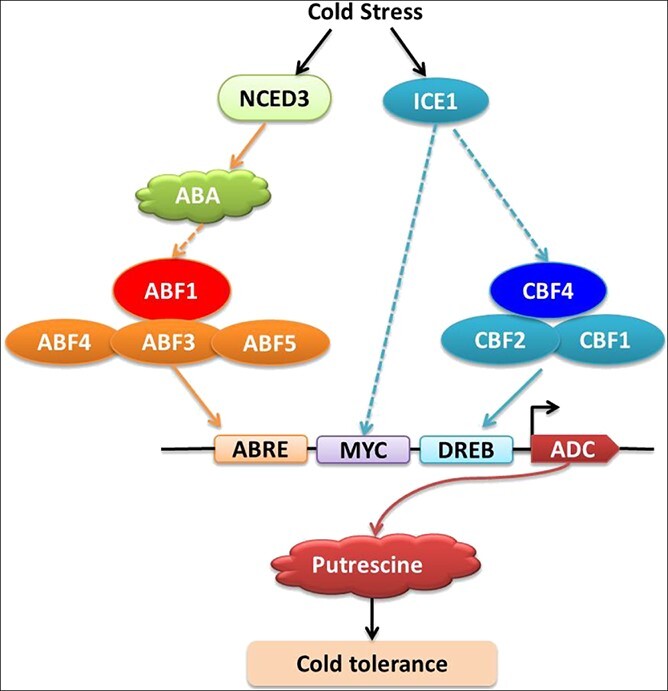
**Model of the cooperative regulation of putrescine synthesis by ABFs and CBFs to promote cold tolerance in melon seedlings.** Cold stress induces *NCED3* expression to promote ABA accumulation and triggers *ABF1* expression; ABF1 then binds directly to the *ADC* promoter. In addition, cold stress also induces *ICE1* and *CBF4* expression; CBF4 can also bind directly to the *ADC* promoter, and ICE1 may act on ADC directly or indirectly through CBFs. Finally, ABF1 and CBF4 cooperatively regulate *ADC* expression to promote Put synthesis, thereby enhancing the cold tolerance of melon seedlings. In addition, CmCBF1/2 and CmABF3/4/5 show at least partial functional redundancy and complementarity with CmABF1 and CmCBF4.

In the current study, we demonstrated that *ADC*-mediated Put synthesis is involved in the cold tolerance of melon seedlings. Furthermore, *ADC* may be an intersection point of the ABA-dependent pathway and the CBF-dependent pathway in melon cold tolerance. In our proposed model (Fig. 7), cold stress induces *NCED3* expression to promote ABA accumulation; it also triggers *ABF1* expression, and ABF1 binds directly to the *ADC* promoter. Furthermore, cold stress induces *ICE1* and *CBF4* expression; CBF4 can also bind directly to the *ADC* promoter, and ICE1 may act on ADC directly or indirectly through CBFs. Finally, ABF1 and CBF4 cooperatively regulate *ADC* expression to promote Put synthesis, thereby enhancing the cold tolerance of melon seedlings. In addition, CmCBF1/2 and CmABF3/4/5 show at least partial functional redundancy and complementarity with CmABF1 and CmCBF4.

## Materials and methods

### Plant materials, growth conditions, and stress treatment

A cold-tolerant genotype, IVF571, was screened according to a preliminary test [61]. At the one-leaf stage, the melon seedlings were transplanted to pots (7 cm × 7 cm) filled with a 3:1 (v/v) mixture of peat and vermiculite. The plants were grown in an intelligent incubator with 70% relative humidity and a 12 h light/12 h dark photoperiod. The day/night temperature was 28°C/18°C, and the light intensity was 500 μmol/(m^2^ s). At the three-leaf stage, the seedlings were treated with low temperatures of 15°C/6°C (12 h light/12 h dark) for 3 d. The third true leaf from the bottom was sampled.

*Nicotiana benthamiana* was grown in a growth chamber at 25°C with 70% relative humidity and a 16 h light/8 h dark photoperiod. Seven-week-old tobacco plants were used for *Agrobacterium*-mediated transient transformation.

### Total RNA extraction and gene transcript abundance analysis by RT-qPCR

The third set of true leaves from melon seedlings were ground into a fine powder in liquid nitrogen. Total RNA was extracted with an Ultrapure RNA kit (CWBIO, Beijing, China). Template cDNA synthesis was performed using PrimeScript RT Master Mix (Takara, Dalian, China) following the manufacturer’s recommendations. RT-qPCR was performed using SYBR Premix Ex Taq II (TaKaRa, Dalian, China) in a 20-μL volume. The amplification program was as follows: one cycle of 30 s at 95°C, followed by 45 cycles of 5 s at 95°C, 15 s at 60°C, and 72°C for 15 s. Relative gene expression levels were calculated using the 2^−ΔΔCt^ method; 18S rRNA from melon was used as the internal control gene. All analyses and error bar calculations were performed using at least three biological replicates. Primer sequences used for RT-qPCR are listed in Table S1.

### Gene identification, cloning, and vector construction

First, we searched the gene names in the melon genomics database (MELONOMICS, http://melonomics.net) and the Cucurbit Genomics Database (CuGenDB, http://cucurbitgenomics.org/). Second, we searched the aforementioned websites for the protein sequences of known crops (Arabidopsis, rice, tomato, and cucumber) (BLASTP). Third, all the genes were further confirmed based on the presence of conserved sequences using DNAMAN6.0. The CDSs of the *CmCBFs* and *CmABFs* and *CmADC* promoter fragments of various lengths were cloned from IVF571 leaves. Fragments used for the VIGS system were cloned from the corresponding full-length CDSs. The gene fragments were cloned into different vectors using the in-fusion method through single or double enzyme restriction sites. The reaction was performed using 5× In-Fusion HD Enzyme Premix (TaKaRa, Dalian, China) in a 10-μL volume with 15 min incubation at 50°C. The fusion plasmids were transformed into *Escherichia coli* DH5α cells to obtain the correct recombinant vector by sequencing. The primer sequences used for cloning are listed in Table S2 in the Supporting Information.

### Promoter sequence and activity analysis

The cloned promoter sequences were submitted to the PlantCARE website (http://bioinformatics.psb.ugent.be/webtools/plantcare/html/) for identification of *cis*-regulatory elements. Promoter activity was analyzed by GUS histological staining as previously described [62].

### Subcellular localization of CmABFs and CmCBFs

The full-length CDSs of *CmABFs* and *CmCBFs* were separately cloned into the *pCAMBIA1300* vector to create four *35S::CmCBF-GFP* and five *35S::CmABF-GFP* fusion constructs, which were subsequently introduced into *Agrobacterium tumefaciens* strain EHA105. The constructs were infiltrated into *N. benthamiana* leaves, and GFP fluorescence in the transgenic leaves was observed using a laser scanning confocal microscope after staining with the nucleus-specific dye 4,6-diamidino-2-phenylindole (DAPI), as previously described by Chen et al. [56]. The primer sequences used for vector construction are listed in Table S3 in the Supporting Information.

### Transactivation assay

Transactivation assays were performed as previously described [63]. In brief, the full-length CDSs of *CmCBFs* and *CmABFs* were separately cloned into the *pGBKT7* vector (BD). The fusion plasmids were introduced into the yeast strain Y2H Gold (TaKaRa, Dalian, China). Transformants were inoculated on SD/−Trp medium. Correct colonies were then transferred to selective SD/−Trp/−His/−Ade medium with X-α-Gal and incubated at 30°C for three days. *pGBKT7* and *pCL-1* vectors were used as negative and positive controls, respectively. The primer sequences used for the assay are listed in Supporting Information Table S4.

### Yeast one-hybrid assay

A yeast one-hybrid (Y1H) assay was performed to determine the binding activity of CmCBFs and CmABFs to *CmADC* promoters in the yeast strain Y1H Gold. The promoter fragments (*CmADC-p1*, *CmADC-p2*, and *CmADC-p3*) were separately inserted into the *pAbAi* vector, and full-length CDSs of *CmABFs* and *CmCBFs* were separately cloned into the *pGADT7* vector. The Y1H assay was performed using the Matchmaker Gold yeast one-hybrid system (TaKaRa, Dalian, China) as previously described [57]. The promoter sequences (*CmADC-p1*, *CmADC-p2*, and *CmADC-p3*) are listed in Supporting Information Table S5. The primer sequences used for the Y1H assay are listed in Supporting Information Table S6.

### GUS activity assay

The *CmADC* promoter sequence (929 bp upstream of the translation start site, listed in Supplementary Table S4) was cloned into the upstream of the GUS reporter gene in the *pBI101* vector to generate a reporter construct. The full-length CDSs of *CmCBFs* and *CmABFs* were separately introduced into the *pRI101* vector to form effector constructs (Figs. 4, 5). The reporter and effector constructs were co-infiltrated into *N. benthamiana* leaves. GUS activity and histochemistry staining were performed as previously described [57]. The infiltration was repeated at least three times independently. The primers used are listed in the Supporting Information Table S7.

### Luciferase reporter assay

The *CmADC* promoter sequence (Table S4) was inserted upstream of the LUC reporter gene in the *pRI101* vector to generate a reporter construct. The effectors were the same as those in the GUS activity assay (Figs. 4, 5). To verify whether these interactions occur *in vivo* in melon, the same infiltrate was injected into cotyledons of ten-day-old melon seedlings. After 48 h, the cotyledons were photographed. The infected tobacco leaves and melon cotyledons were measured using an *in vivo* fluorescence plant imaging system (LB985, Berthold, Germany) as previously described [56]. The primers used are listed in Supporting Information Table S7.

### VIGS system in melon seedlings

Full-length *CmCBF1*, *CmCBF2*, and *CmCBF4* and approximately 300-bp fragments of six other genes (*CmABF1*, *CmABF3*, *CmABF4*, *CmABF5*, *CmADC* and *CmNCED3*) were introduced into the *pTRV2* vector. The detailed process has been described previously [58]. Each gene construct was used to infect approximately 45 plants. Plants were randomly assigned to one of three groups for sampling at 0, 1, and 3 days after low-temperature treatment, and each plant was sampled independently. *TRV2* vector-specific primers were designed and amplified by PCR to determine whether the infection was successful using 1% agarose gel electrophoresis (Fig. S1). Simultaneously, the expression of the gene in leaves of VIGS plants was detected using RT-qPCR, and plants that showed transcript levels <50% of those of the control plants were used for cold treatment. The primers used are listed in Supporting Information Table S8.

Exogenous Put was applied to the silenced plants to test whether the phenotype could be recovered and to determine whether Put was involved. The silenced plants were sprayed with 1 mM Put 12 h before the initiation of cold treatment [64].

### Determination of chlorophyll fluorescence and ion leakage

Melon seedlings were dark-adapted for 30 min to measure the maximum photochemical efficiency of photosystem II (Fv/Fm) with a Dual PAM-100 fluorometer (Heinz Walz, Germany) as previously described [61]. The value of ion leakage was measured after cold treatment (15°C/6°C) for 0, 1, or 3 days as previously described [61].

### Determination of Put by HPLC

Each leaf sample (0.3 g) was ground with 5 mL 5% (v/v) perchloric acid and then benzoylated for determination. Put was measured by high-performance liquid chromatography (HPLC) as detailed by Song et al [14].

### Determination of ABA content by ELISA

Each leaf sample (0.3 g) was ground with 5 mL 100 mM phosphate buffer (pH 7.4), and the supernatant was collected after centrifugation at 8000 rpm for 20 min. The ABA level was then quantified using an ELISA kit (MBBiology, Jiangsu, China) according to the protocol provided by the manufacturer [17].

## Statistical analysis

All samples were assessed independently at least three times, and all data are presented as the mean ± SD. The statistical analysis was performed with the SPSS 18.0 software package. Independent *t*-tests and one-way ANOVA were used to analyze the data, and Duncan’s multiple comparisons were used for sample comparisons at a significance level of *P* < 0.05 or *P* < 0.01. Significance is indicated by asterisks (*P < 0.05, **P < 0.01) or different letters.

## Acknowledgements

This work was supported by the China Agriculture Research System of MOF and MARA. Funder ID CARS-25. We thank Professor Huaisong Wang (Institute of Vegetables and Flowers, Chinese Academy of Agricultural Sciences) for providing melon seeds. We thank Professor Aide Wang and Professor Yue Ma (Shenyang Agriculture University) for their experimental technical assistance.

## Author contributions

HYQ and TL designed this project and revised the manuscript.

ML performed most of the experiments and wrote the manuscript.

XYD and GG helped to perform Y1H, GUS activity, and luciferase reporter assays.

## Conflict of interest statement

The authors declare that they have no conflict of interest.

## Supplementary data

Supplementary data are available at *Horticulture Research* online.

## Supplementary Material

Web_Material_uhac002
