# Supplementary material for: CmABF1 and CmCBF4 cooperatively regulate putrescine synthesis to improve cold tolerance of melon seedlings
Source: Hortic Res. 2022 Feb 11;9:uhac002. doi: 10.1093/hr/uhac002 (PMC9016860; doi:10.1093/hr/uhac002)
Supplement: Web_Material_uhac002 [file web_material_uhac002.pdf]

Supporting Information

Table S1 Primers used for gene expression

**Primer name**	**Sequence (5'-3')**
CmICE1-ex-F	GATTTAGGCGGTGAAAATGGT
CmICE1-ex-R	GAAAGTCGGAGTTGTTTAAGGTGA
CmCBF1-ex-F	ATGGATTCTTTTTCAAATTTTTATG
CmCBF1-ex-R	GTTTCTCCTCCTGACTCCTCTGTAT
CmCBF2-ex-F	GTCTTCCCATCGTTCCACTCTT
CmCBF2-ex-R	GCTCTCTAACTTCACAAACCCACT
CmCBF3-ex-F	CCCTCATCGTTGTGTTCTTTA
CmCBF3-ex-R	CCCAATCGAATTCCCTCTTC
CmCBF4-ex-F	GAATGAGGGTAGAGATGAGG
CmCBF4-ex-R	GGAGGAGAAAGAAGTAGTCC
CmADC-ex-F	ACATTGGAGGTGGTCAGG
CmADC-ex-R	AGCCGAAAGGTTCTGATA
CmNCED3-ex-F	CGGAATGGTGAACCGAAATCTACTT
CmNCED3-ex-R	TTCTCGTCGTGAACGAAGGCTAAG
CmABF1-ex-F	AGTTTGGCTCTTGGGATGTTG
CmABF1-ex-R	AATGCCCGTGCCTTGAAT
CmABF2-ex-F	CTTCAGTGTCTCCTGTGCCTTAC
CmABF2-ex-R	ATCATTCTCCGTTGCCTCCT
CmABF3-ex-F	CCAATCAAACATCCCTACAACG
CmABF3-ex-R	GGACATCCATCACAGCACCAC
CmABF4-ex-F	ATCAAAAGCGACAAGAATCAAG
CmABF4-ex-R	TAAAGGGTCAAAACAACACAACT
CmABF5-ex-F	GCGAAAAGAGACAGAAACCAAC
CmABF5-ex-R	TCCTCAAAGTGACAACAAAAACAT

Table S2 Primers used for gene clone

Primer name	Sequence (5'-3')	Length
CmCBF1-full-F	ATGGATTCTTTTTCAAATTTTTATGAAG	639 bp
CmCBF1-full-R	TTAATAGCTCCATAAGGACACGTCAG	
CmCBF2-full-F	ATGGAACTTTCCGATGCT	606 bp
CmCBF2-full-R	TCAAATGGAGTAACTCCA	
CmCBF3-full-F	ATGCCTTCCTCGTCTTCCAGCTC	609 bp
CmCBF3-full-R	TCACTCATGACTCCATAACGAC	
CmCBF4-full-F	ATGGATTGGTTCGCTCAA	690 bp
CmCBF4-full-R	CTAAAAGCTCCACAATGA	
CmABF1-full-F	ATGAATTTCAGAAACTTTGAGGATATC	1230 bp
CmABF1-full-R	CTACCATGGGCCAGTCAGTGTT	
CmABF2-full-F	GGATCCGGTACTGGTTCTTATT	1236 bp
CmABF2-full-R	TATTGTCCTCCAAAATCACCAT	
CmABF3-full-F	ATGGGGATTCAAACTATGGGGT	966 bp
CmABF3-full-R	TTAGAACGGCGCTGATGATGTT	
CmABF4-full-F	ATGGGGATTCAGACGATGGG	813 bp
CmABF4-full-R	TTAGAAGGAAGCTGAACTAGTTCTACG	
CmABF5-full-F	ATGGAGAATTCTCAATTGGAAG	993 bp
CmABF5-full-R	TCAGCCTGCACAGTTTTTTAT	
CmADC-pro-F	AGCTACATATAAATGCCACGTAAG	929 bp
CmADC-pro-R	CTCCGACGACGGATCAAGAAAT	
CmADC-p1-F	AGCTACATATAAATGCCACGTAAG	168 bp
CmADC-p1-R	AAGGAGTGAGATTCTATTTTTCAT	
CmADC-p2-F	GGGTCATAAACTACCGGGTT	172 bp
CmADC-p2-R	AGAAAACGCGGAGAGAAATG	
CmADC-p3-F	GGCGGTGGAAATAGCTACTCTTT	201 bp
CmADC-p3-R	CTCCGACGACGGATCAAGAAAT	
CmADC-VIGS-F	TTCGTCCTGTCGTTGGTATGCG	321 bp
CmADC-VIGS-R	ACTTCGACCCGTCATAGTC	
CmNCED3-VIGS-F	ATGGGATGGTTCATGCGG	342bp
CmNCED3-VIGS-R	CGAAATCGAACCGGCCAAC	
CmICE1-VIGS-F	ATCCTTCCCACCAAGATTTC	325 bp
CmICE1-VIGS-R	GGAAGAGACGAAATGCCAGT	
CmABF1-VIGS-F	GAGTCTCAGGCTGTAACATC	299 bp
CmABF1-VIGS-R	CAGTTAAATGGCCGCTCC	
CmABF2-VIGS-F	CCATTAGCGCGACAATCA	347 bp
CmABF2-VIGS-R	GTTTGCTGCCTCTGTTGC	
CmABF3-VIGS-F	TGCGGGCCCTGTTGTTGAA	279 bp
CmABF3-VIGS-R	ATCATGTCTTCAGGCGCTCC	
CmABF4-VIGS-F	CTCTCCAACGTCAGGCCAGT	300 bp
CmABF4-VIGS-R	CTGACAGTGTGCCTAGTG	
CmABF5-VIGS-F	GGTGAAAGCTGGAGTTGTTC	312bp
CmABF5-VIGS-R	GGACCATCGATTATCCTCTTC	

Table S3 Primers used for subcellular localization of *CmABFs* and *CmCBFs*

Primer name	Sequence (5'-3')
CmABF1-1300-BamHI-F	GGTACCCGGGGATCC ATGAATTTCAGAAACTTTGAGGATAT
CmABF1-1300-BamHI-R	GACTCTAGAGGATCC CCATGGGCCAGTCAGTGTTC
CmABF2-1300-BamHI-F	GGTACCCGGGGATCC GGATCCGGTACTGGTTCTTATT
CmABF2-1300-BamHI-R	GACTCTAGAGGATCC CCATGGGCCAGTCTGTGTTC
CmABF3-1300-BamHI-F	GGTACCCGGGGATCC ATGGGGATTCAAACTATGGGGT
CmABF3-1300-BamHI-R	GACTCTAGAGGATCC GAACGGCGCTGATGATGTTC
CmABF4-1300-BamHI-F	GGTACCCGGGGATCC ATGGGGATTCAGACGATGGG
CmABF4-1300-BamHI-R	GACTCTAGAGGATCC GAAGGAAGCTGAACTAGTTCTACGC
CmABF5-1300-BamHI-F	GGTACCCGGGGATCC ATGGAGAATTCTCAATTGGAAG
CmABF5-1300-BamHI-R	GACTCTAGAGGATCC GCCTGCACAGTTTTTTATGTT
CmCBF1-1300-BamHI-F	GGTACCCGGGGATCC ATGGATTCTTTTTCAAATTTTTATGAAG
CmCBF1-1300-BamHI-R	GACTCTAGAGGATCC ATAGCTCCATAAGGACACGTCAGAG
CmCBF2-1300-BamHI-F	GGTACCCGGGGATCC ATGGAACTTTCCGATGCTTCTT
CmCBF2-1300-BamHI-R	GACTCTAGAGGATCC AATGGAGTAACTCCACAACGACAT
CmCBF3-1300-BamHI-F	GGTACCCGGGGATCC ATGCCTTCCTCGTCTTCCAG
CmCBF3-1300-BamHI-R	GACTCTAGAGGATCC CTCATGACTCCATAACGACAATTC
CmCBF4-1300-BamHI-F	GGTACCCGGGGATCC ATGGATTGGTTCGCTCAATTT
CmCBF4-1300-BamHI-R	GACTCTAGAGGATCC CTAAAAGCTCCACAATGA

Note: The underlined part indicates the restriction site with a small part of vector sequence

Table S4 Primers used for transactivation assays

Primer name	Sequence (5'-3')
CmABF1-pGBKT7-EcoRI-F	ATGGCCATGGAGGCCGAATTC ATGAATTTCAGAAACTTTGAGGATATC
CmABF1-pGBKT7-PstI-R	TAGTTATGCGGCCGCTGCAGG CTACCATGGGCCAGTCAGTGTT
CmABF2-pGBKT7-EcoRI-F	ATGGCCATGGAGGCCGAATTC ATGAATTTCAAAGATTTTGGGAAT
CmABF2-pGBKT7-PstI-R	TAGTTATGCGGCCGCTGCAGG TCACCATGGGCCAGTCTGTG
CmABF3-pGBKT7-EcoRI-F	ATGGCCATGGAGGCCGAATTC ATGGGGATTCAAACTATGGGGT
CmABF3-pGBKT7-PstI-R	TAGTTATGCGGCCGCTGCAGG TTAGAACGGCGCTGATGATGTT
CmABF4-pGBKT7-EcoRI-F	ATGGCCATGGAGGCCGAATTC ATGGGGATTCAGACGATGGG
CmABF4-pGBKT7-PstI-R	TAGTTATGCGGCCGCTGCAGG TTAGAAGGAAGCTGAACTAGTTCTACG
CmABF5-pGBKT7-EcoRI-F	ATGGCCATGGAGGCCGAATTC ATGGAGAATTCTCAATTGGAAG
CmABF5-pGBKT7-PstI-R	TAGTTATGCGGCCGCTGCAGG TCAGCCTGCACAGTTTTTTAT
CmCBF1-pGBKT7-EcoRI-F	ATGGCCATGGAGGCCGAATTC ATGGATTCTTTTTCAAATTTTTATGAAG
CmCBF1-pGBKT7-PstI-R	TAGTTATGCGGCCGCTGCAGG TTAATAGCTCCATAAGGACACGTCAG
CmCBF2-pGBKT7-EcoRI-F	ATGGCCATGGAGGCCGAATTC ATGGAACTTTCCGATGCT
CmCBF2-pGBKT7-PstI-R	TAGTTATGCGGCCGCTGCAGG TCAAATGGAGTAACTCCA
CmCBF3-pGBKT7-EcoRI-F	ATGGCCATGGAGGCCGAATTC ATGCCTTCCTCGTCTTCCAGCTC
CmCBF3-pGBKT7-PstI-R	TAGTTATGCGGCCGCTGCAGG TCACTCATGACTCCATAACGAC
CmCBF4-pGBKT7-EcoRI-F	ATGGCCATGGAGGCCGAATTC ATGGATTGGTTCGCTCAA
CmCBF4-pGBKT7-PstI-R	TAGTTATGCGGCCGCTGCAGG CTAAAAGCTCCACAATGA

Note: The underlined part indicates the restriction site with a small part of vector sequence

Table S5 The promoter sequence of *CmADC*

The promoter sequence of CmADC
AGCTACATATAAATGCCACGTAAGCAAGGATAACAAGAAGGGCGAAGCCTCGGGAGATGTA<br>TAGGGTAATGGAGGGGTCCCGCATGAAAGTAGGCCACGCGGCAACCCGACGCTGATTAGTC<br>CATTAATTTTGAAGAAAGAAAAATGAAAAATAGAATCTCACTCCTTTTTTTTTTTTTTTTTCC<br>CTTTCCTTCTTTTCATAAAAATAAGTTAAGATTTTAAAAAAATAATAATAAAAACCGCCAGA<br>AATATTATCGGGTCATAAACTACCGGGTTAATGGGTGGCTGCTATTAAGATCCGAACCGAAC<br>CCGATTCATATTCTCCTATAAAAGGACGCTTGGTAATGCATGCAAATCTCGCTCATTCTAACC<br>TCGTTTAAACGCATTCCGTCGTCTCCGACGGCTCTACATTTCTCTCCGCGTTTTCTCCGTTAG<br>GTTTCCGTTTCTTCCTTCATCTTCTTCTTTTTCTCCGCTTCTTTGCCCGGAAAATTCTCCTTCC<br>TTCCATTCCCGATTTCTCTTTTTTTTTATTCTTTTTTTTTTTAACATAAATTTTCAATTCCGTTC<br>TTTGAATTCGTTTCCAGATCTGCATGGTGAAGAACAGTTTCGGTGTTTAAGGCTTTTGGTGT<br>GTTTTTTTAATTTCATTTTTTGTTTCTTTGTTCTTGTGAAATTCTTCTTCTGACCGGATCGCGG<br>TGGGATATTGCAAGTGTATAGGTGATCGTGGTAAATTTGGCGGTGGAAATAGCTACTCTTTCT<br>GCTTCTTTTGGGGGGGTAGCCGGGGCCTCGGCCTCGGCGGGTTTTAAAGCCCCCACTTGCA<br>CAAAACTCTGAAACTTCCATTACTTTTTCTTAACAACTTCTCTAACTACGACTCTCTTCTTTC<br>TTTAATTTTTCCATTTTCTTCTTTTCATTCATTTCTTGATCCGTCGTCGGAG

Note: The sequence of gray markers is *CmADC-p1*, *CmADC-p2* and *CmADC-p3* in turn

DREB *cis* acting elements are marked as yellow;

AREB *cis* acting elements are marked as green.

Table S6 Primers used for Yeast one-hybrid assays

Primer name	Sequence (5’-3’)
CmABF1-pGADT7-SmaI-F	CAGTGAATTCCACCC ATGAATTTCAGAAACTTTGAGGATATC
CmABF1-pGADT7-SmaI-R	TATCGATGCCCACCC CTACCATGGGCCAGTCAGTGTT
CmABF2-pGADT7-SmaI-F	CAGTGAATTCCACCC GGATCCGGTACTGGTTCTTATT
CmABF2-pGADT7-SmaI-R	TATCGATGCCCACCC TATTGTCCTCCAAAATCACCAT
CmABF3-pGADT7-SmaI-F	CAGTGAATTCCACCC ATGGGGATTCAAACTATGGGGT
CmABF3-pGADT7-SmaI-R	TATCGATGCCCACCC TTAGAACGGCGCTGATGATGTT
CmABF4-pGADT7-SmaI-F	CAGTGAATTCCACCC ATGGGGATTCAGACGATGGG
CmABF4-pGADT7-SmaI-R	TATCGATGCCCACCC TTAGAAGGAAGCTGAACTAGTTCTACG
CmABF5-pGADT7-SmaI-F	CAGTGAATTCCACCC ATGGAGAATTCTCAATTGGAAG
CmABF5-pGADT7-SmaI-R	TATCGATGCCCACCC TCAGCCTGCACAGTTTTTTAT
CmCBF1-pGADT7-SmaI-F	CAGTGAATTCCACCC ATGGATTCTTTTTCAAATTTTTATGAAG
CmCBF1-pGADT7-SmaI-R	TATCGATGCCCACCC TTAATAGCTCCATAAGGACACGTCAG
CmCBF2-pGADT7-SmaI-F	CAGTGAATTCCACCC ATGGAACTTTCCGATGCT
CmCBF2-pGADT7-SmaI-R	TATCGATGCCCACCC TCAAATGGAGTAACTCCA
CmCBF3-pGADT7-SmaI-F	CAGTGAATTCCACCC ATGCCTTCCTCGTCTTCCAGCTC
CmCBF3-pGADT7-SmaI-R	TATCGATGCCCACCC TCACTCATGACTCCATAACGAC
CmCBF4-pGADT7-SmaI-F	CAGTGAATTCCACCC ATGGATTGGTTCGCTCAA
CmCBF4-pGADT7-SmaI-R	TATCGATGCCCACCC CTAAAAGCTCCACAATGA
CmADC-pro-pAbAi-SmaI-F	TCGAGCTCGGTACCC AGCTACATATAAATGCCACGTAAG
CmADC-pro-pAbAi-XhoI-R	GAGCACATGCCTCGA CTCCGACGACGGATCAAGAAAT
CmADC-p1-pAbAi-SmaI-F	TCGAGCTCGGTACCC AGCTACATATAAATGCCACGTAAG
CmADC-p1-pAbAi-XhoI-R	GAGCACATGCCTCGA AAGGAGTGAGATTCTATTTTTCAT
CmADC-p2-pAbAi-SmaI-F	TCGAGCTCGGTACCC GGGTCATAAACTACCGGGTT
CmADC-p2-pAbAi-XhoI-R	GAGCACATGCCTCGA AGAAAACGCGGAGAGAAATG
CmADC-p3-pAbAi-SmaI-F	TCGAGCTCGGTACCC GGCGGTGGAAATAGCTACTCTTT
CmADC-p3-pAbAi-XhoI-R	GAGCACATGCCTCGA CTCCGACGACGGATCAAGAAAT

Note: The underlined part indicates the restriction site with a small part of vector sequence

Table S7 Primers used for GUS activity assay and luciferase reporter assay

Primer name	Sequence (5’-3’)
CmABF1-PRI101-NdeI-F	TCTTCACTGTTGATACATATG ATGAATTTCAGAAACTTTGAGGATATC
CmABF1-PRI101-EcoRI-R	AGAGTTGTTGATTCAGAATTC CTACCATGGGCCAGTCAGTGTT
CmABF2-PRI101-NdeI-F	TCTTCACTGTTGATACATATG ATGAATTTCAAAGATTTTGGGAAT
CmABF2-PRI101-EcoRI-R	AGAGTTGTTGATTCAGAATTC TCACCATGGGCCAGTCTGTG
CmABF3-PRI101-NdeI-F	TCTTCACTGTTGATACATATG ATGGGGATTCAAACTATGGGGT
CmABF3-PRI101-EcoRI-R	AGAGTTGTTGATTCAGAATTC TTAGAACGGCGCTGATGATGTT
CmABF4-PRI101-NdeI-F	TCTTCACTGTTGATACATATG ATGGGGATTCAGACGATGGG
CmABF4-PRI101-EcoRI-R	AGAGTTGTTGATTCAGAATTC TTAGAAGGAAGCTGAACTAGTTCTACG
CmABF5-PRI10-NdeI-F	TCTTCACTGTTGATACATATG ATGGAGAATTCTCAATTGGAAG
CmABF5-PRI101-EcoRI-R	AGAGTTGTTGATTCAGAATTC TCAGCCTGCACAGTTTTTTAT
CmCBF1-PRI101-NdeI-F	TCTTCACTGTTGATACATATG ATGGATTCTTTTTCAAATTTTTATGAAG
CmCBF1-PRI101-EcoRI-R	AGAGTTGTTGATTCAGAATTC TTAATAGCTCCATAAGGACACGTCAG
CmCBF2-PRI101-NdeI-F	TCTTCACTGTTGATACATATG ATGGAACTTTCCGATGCT
CmCBF2-PRI101-EcoRI-R	AGAGTTGTTGATTCAGAATTC TCAAATGGAGTAACTCCA
CmCBF3-PRI101-NdeI-F	TCTTCACTGTTGATACATATG ATGCCTTCCTCGTCTTCCAGCTC
CmCBF3-PRI101-EcoRI-R	AGAGTTGTTGATTCAGAATTC TCACTCATGACTCCATAACGAC
CmCBF4-PRI101-NdeI-F	TCTTCACTGTTGATACATATG ATGGATTGGTTCGCTCAA
CmCBF4-PRI101-EcoRI-R	AGAGTTGTTGATTCAGAATTC CTAAAAGCTCCACAATGA
CmADC-pro-PBI101-SalI-F	CTTGCATGCCTGCAGGTCGAC AGCTACATATAAATGCCACGTAAG
CmADC- pro-PBI101-SmaI-R	AAGGGACTGACCACCCGG CTCCGACGACGGATCAAGAAAT
CmADC-pro-pRI-mini35S-HindIII-F	ACGACGGCCAGTGCCAAGCTT AGCTACATATAAATGCCACGTAAG
CmADC-pro-PRI101-mini35S-SmaI-R	ATTCGGATCCGGTACCCCC CTCCGACGACGGATCAAGAAAT

Note: The underlined part indicates the restriction site with a small part of vector sequence

Table S8 Primers used for VIGS assay

Primer name	Sequence (5’-3’)
CmICE1-pTRV2-EcoRI-F	<u>TAAGGTTACCGAATTC</u> ATCCTTCCCACCAAGATTTC
CmICE1-pTRV2-BamHI-R	<u>GCTCGGTACCGGATCC</u> GGAAGAGACGAAATGCCAGT
CmADC-pTRV2-EcoRI-F	<u>TAAGGTTACCGAATTC</u> TTCGTCCTGTCGTTGGTATGCG
CmADC-pTRV2-BamHI-R	<u>GCTCGGTACCGGATCC</u> ACTTCGACCCGTCATAGTC
CmNCED3-pTRV2-EcoRI-F	<u>AAGGTTACCGAATTC</u> ATGGGATGGTTCATGCGG
CmNCED3-pTRV2-BamHI-R	<u>CTCGGTACCGGATCC</u> CGAAATCGAACCGGCCAAC
CmABF1-pTRV2-EcoRI-F	<u>AAGGTTACCGAATTC</u> GAGTCTCAGGCTGTAACATC
CmABF1-pTRV2-BamHI-R	<u>CTCGGTACCGGATCC</u> CAGTTAAATGGCCGCTCC
CmABF2-pTRV2-EcoRI-F	<u>AAGGTTACCGAATTC</u> CCATTAGCGCGACAATCA
CmABF2-pTRV2-BamHI-R	<u>CTCGGTACCGGATCC</u> GTTTGCTGCCTCTGTTGC
CmABF3-pTRV2-EcoRI-F	<u>AAGGTTACCGAATTC</u> TGCGGGCCCTGTTGTTGAA
CmABF3-pTRV2-BamHI-R	<u>CTCGGTACCGGATCC</u> ATCATGTCTTCAGGCGCTCC
CmABF4-pTRV2-EcoRI-F	<u>AAGGTTACCGAATTC</u> CTCTCCAACGTCAGGCCAGT
CmABF4-pTRV2-BamHI-R	<u>CTCGGTACCGGATCC</u> CTGACAGTGTGCCTAGTG
CmABF5-pTRV2-EcoRI-F	<u>AAGGTTACCGAATTC</u> GGTGAAAGCTGGAGTTGTTC
CmABF5-pTRV2-BamHI-R	<u>CTCGGTACCGGATCC</u> GGACCATCGATTATCCTCTTC
CmCBF1-pTRV2-EcoRI-F	<u>TAAGGTTACCGAATTC</u> ATGGATTCTTTTTCAAATTTTTATGAAG
CmCBF1-pTRV2-BamHI-R	<u>GCTCGGTACCGGATCC</u> TTAATAGCTCCATAAGGACACGTCAG
CmCBF2-pTRV2-EcoRI-F	<u>TAAGGTTACCGAATTC</u> ATGGAACTTTCCGATGCT
CmCBF2-pTRV2-BamHI-R	<u>GCTCGGTACCGGATCC</u> TCAAATGGAGTAACTCCA
CmCBF3-pTRV2-EcoRI-F	<u>TAAGGTTACCGAATTC</u> ATGCCTTCCTCGTCTTCCAGCTC
CmCBF3-pTRV2-BamHI-R	<u>GCTCGGTACCGGATCC</u> TCACTCATGACTCCATAACGAC
CmCBF4-pTRV2-EcoRI-F	<u>TAAGGTTACCGAATTC</u> ATGGATTGGTTCGCTCAA
CmCBF4-pTRV2-BamHI-R	<u>GCTCGGTACCGGATCC</u> CTAAAAGCTCCACAATGA
pTRV2-test-F	CGTGCTGCGTCAAGGTGGGTCT
pTRV2-test-R	CAGTCGATCACTATTGCGTCTGGTT

Note: The underlined part indicates the restriction site with a small part of vector sequence

Table S9 Detailed information of *CmCBFs* and *CmABFs* in melon genome

Genes name	Genes ID	Accession number	Chr	Location &Direction	Amino acids	pI	Mw(Da)
CmCBF1	MELO3C006869	AMK37721	Chr06	6858149 .. 6858787(-)	212	5.23	23897.75
CmCBF2	MELO3C005367	MZ402513	Chr09	20926191 .. 20926796(-)	201	5.54	22434.24
CmCBF3	MELO3C009442	AMK37722	Chr04	32101447 .. 32102261(+)	202	6.91	22484.57
CmCBF4	MELO3C005629	MZ402514	Chr09	23093651 .. 23094747(-)	229	4.66	25788.50
CmABF1	MELO3C018458	MZ389323	Chr01	423061 .. 427336 (-)	409	9.45	44947.74
CmABF2	MELO3C010850	MZ402509	Chr03	30414454 .. 30418140 (+)	411	9.47	44221.57
CmABF3	MELO3C019925	MZ402510	Chr03	22105035 .. 22111047 (-)	321	8.56	35886.47
CmABF4	MELO3C015611	MZ402511	Chr02	3293621 .. 3297954 (-)	270	9.28	30371.30
CmABF5	MELO3C021421	MZ402512	Chr11	29589884 .. 29592172 (+)	330	6.46	37541.53

Note: Genes ID Chr, chromosome; MW, molecular weight; pI, isoelectric point.

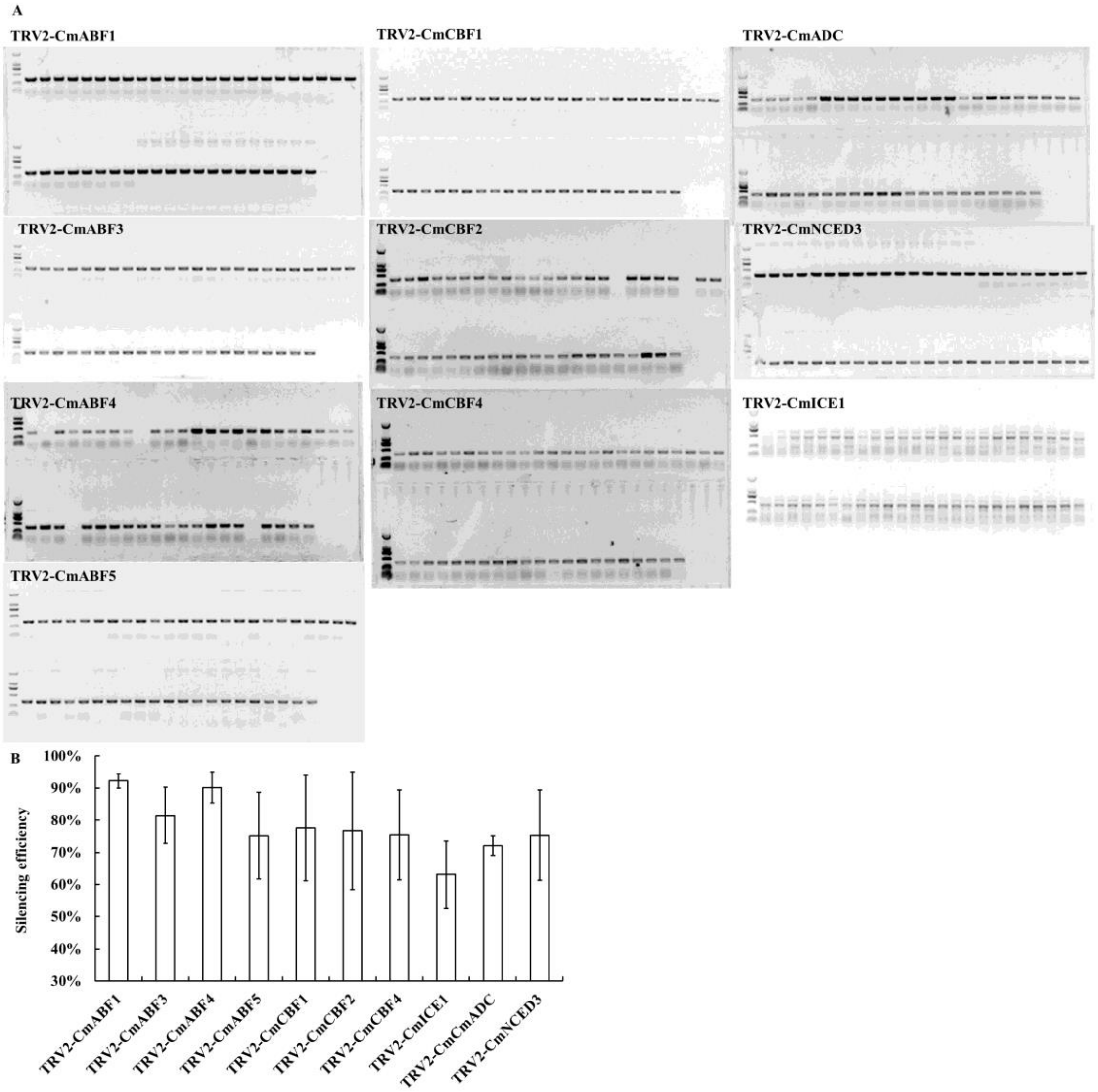

Fig.S1 The infection of TRV virus was detected with 1% agarose gel (A) and RT-qPCR (**B**). The RT-PCR detection of RNA2 of TRV in melon leaves from the whole silencing seedlings (about 45 plants for each gene). There was a band at 368bp, which proved that TRV was successfully infected and the related genes were silenced. About 15 infected seedlings were sampled to detect the silencing efficiency before treatment, as revealed by RT-qPCR.

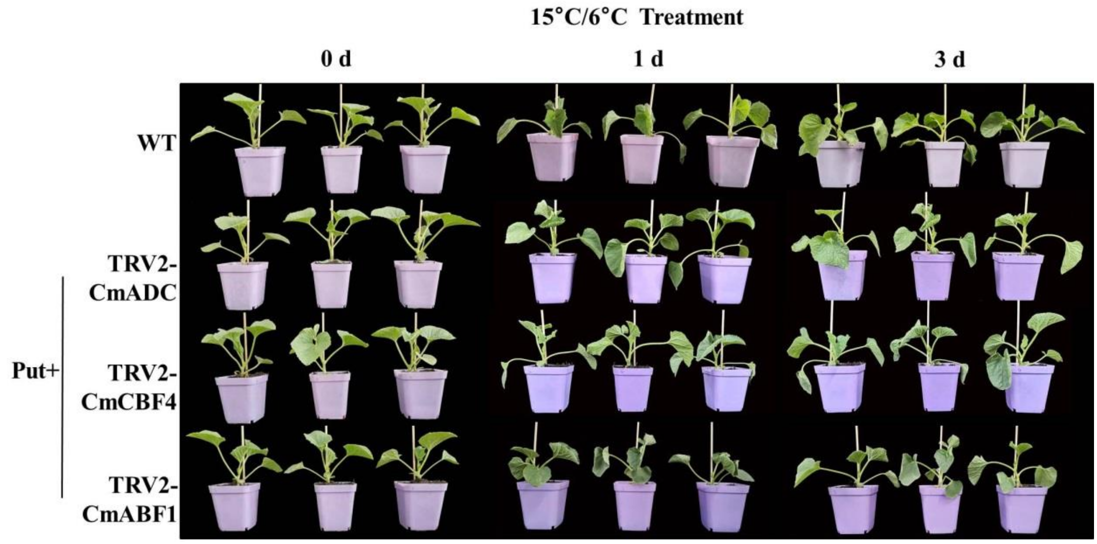

Fig.S2 Exogenous application of Put improved silenced plants cold tolerance.

A Plant phenotype, **B** Fv/Fm. Exogenous Put was applied to the silenced plants to further test whether the phenotype can be recovered so as to see Put is involved. The silenced plants were sprayed with 1 mM of Put 12 h before cold treatment initiated. Independent t-test was used to analyze the difference between silenced plants and control plants (WT). Significant differences are marked with asterisks (**P < 0.01; *P < 0.05). Error bars are shown with the three biological replicates.

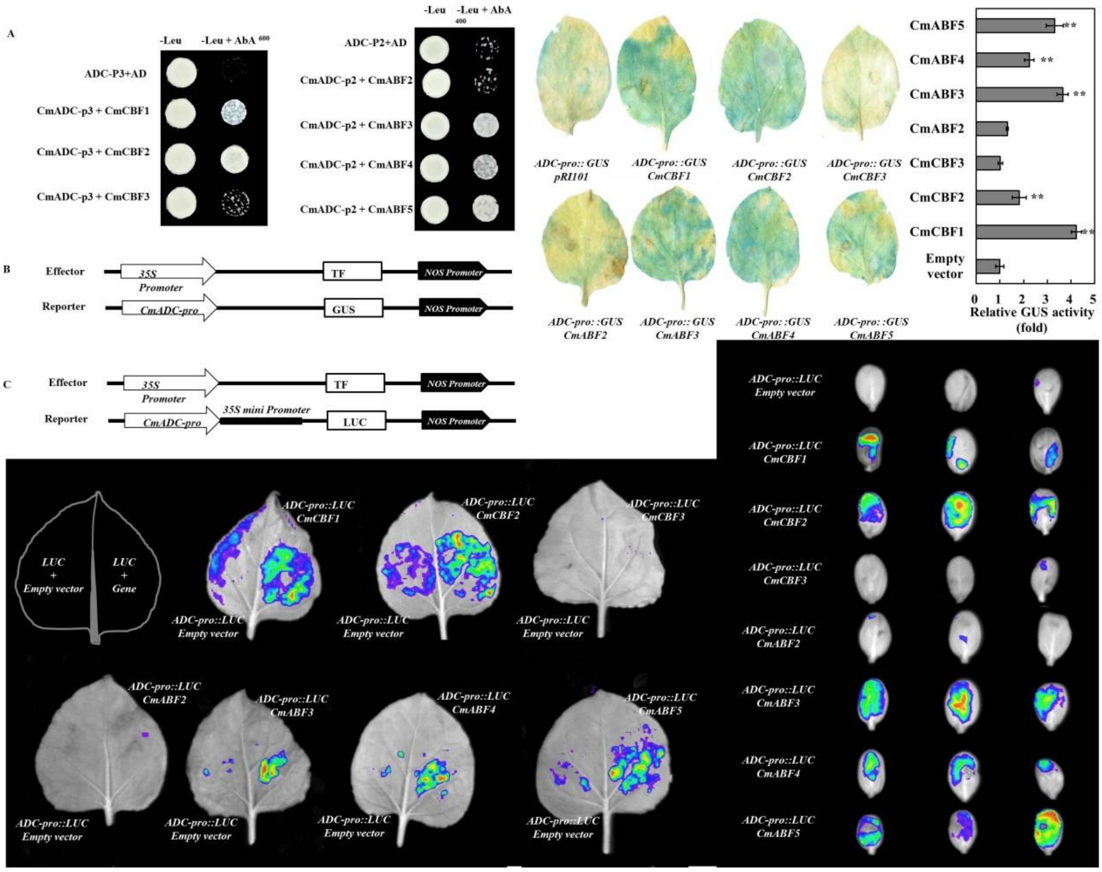

Fig.S3 Other CmCBFs and CmABFs (excluding CmCBF4 and CmABF1) also can directly bind to the promoter of *CmADC*. **A** Y1H analysis of CmCBFs and CmABFs binding to the *CmADC* promoters. The growth status of transformed yeasts on two medium and normal yeast growth on defective medium containing the antibiotic Aureobasidin A indicates CmCBFs and CmABFs can bind to the promoter of *CmADC*. **B** GUS activity assays analysis of CmCBFs and CmABFs binding to the *CmADC* promoter. The histochemical analysis of *GUS* gene activity in tobacco leaves. The staining level represents the abundance of GUS protein in the leaves. Relative GUS activity increasing indicates transcriptional regulation is activated. Independent t-test was used to analyze the difference between silenced plants and control plants (WT). Significant differences are marked with asterisks (**P < 0.01; *P < 0.05). Error bars are shown with the three biological replicates. **C** Luciferase reporter assay showing the *in vivo* binding of CmCBFs and CmABFs to the *CmADC* promoter. The infected tobacco and melon cotyledons were measured by a living fluorescence imager. The fluorescence intensity is stronger than control (empty vector) means that TF interacts with promoter, and activates the gene expression.

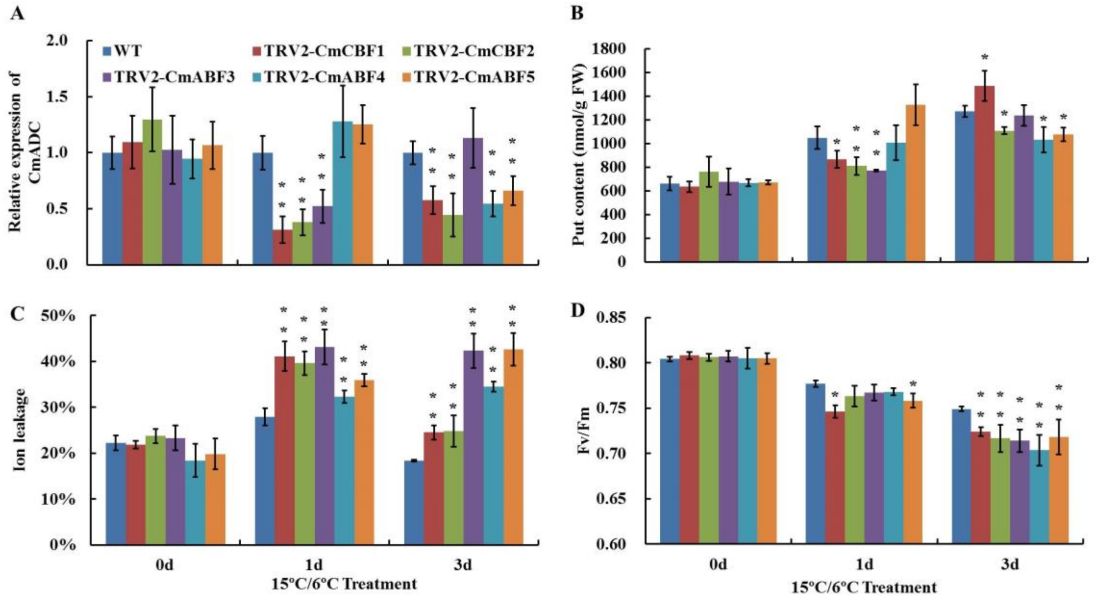

Fig.S4 VIGS of *CmCBF* and *CmABF* (excluding *CmCBF4* and *CmABF1*) resulted in the decrease of Put content and cold tolerance in melon seedlings. *CmADC* expression (**A**), Put accumulation (**B**), ion leakage (**C**), and Fv/Fm (**D**) of *CmCBF4* and *CmABF1* silenced seedlings under cold treatment (15°C/6°C). Independent t-test was used to analyze the difference between silenced plants and control plants (WT). Significant differences are marked with asterisks (**P < 0.01; *P < 0.05). Error bars are shown with the three biological replicates.

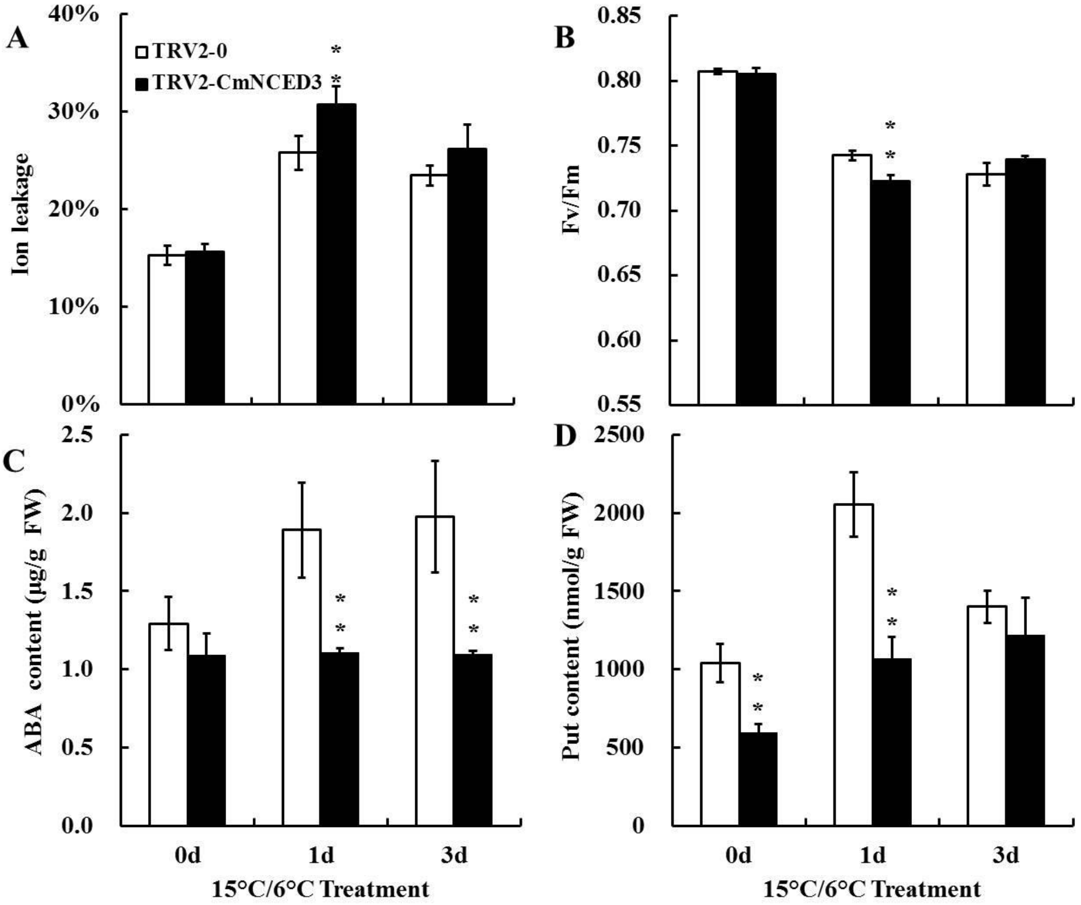

Fig.S5 VIGS of *CmNCED3* resulted in the decrease of Put content and cold tolerance in melon seedlings. Ion leakage (**A**), Fv/Fm (**B**), ABA content (**C**) and Put content (**D**) of melon seedlings under low temperature treatment (15°C/6°C). Independent t-test was used to analyze the difference between silenced plants and control plants (WT). Significant differences are marked with asterisks (**$P < 0.01$; *$P < 0.05$). Error bars are shown with the three biological replicates.

.

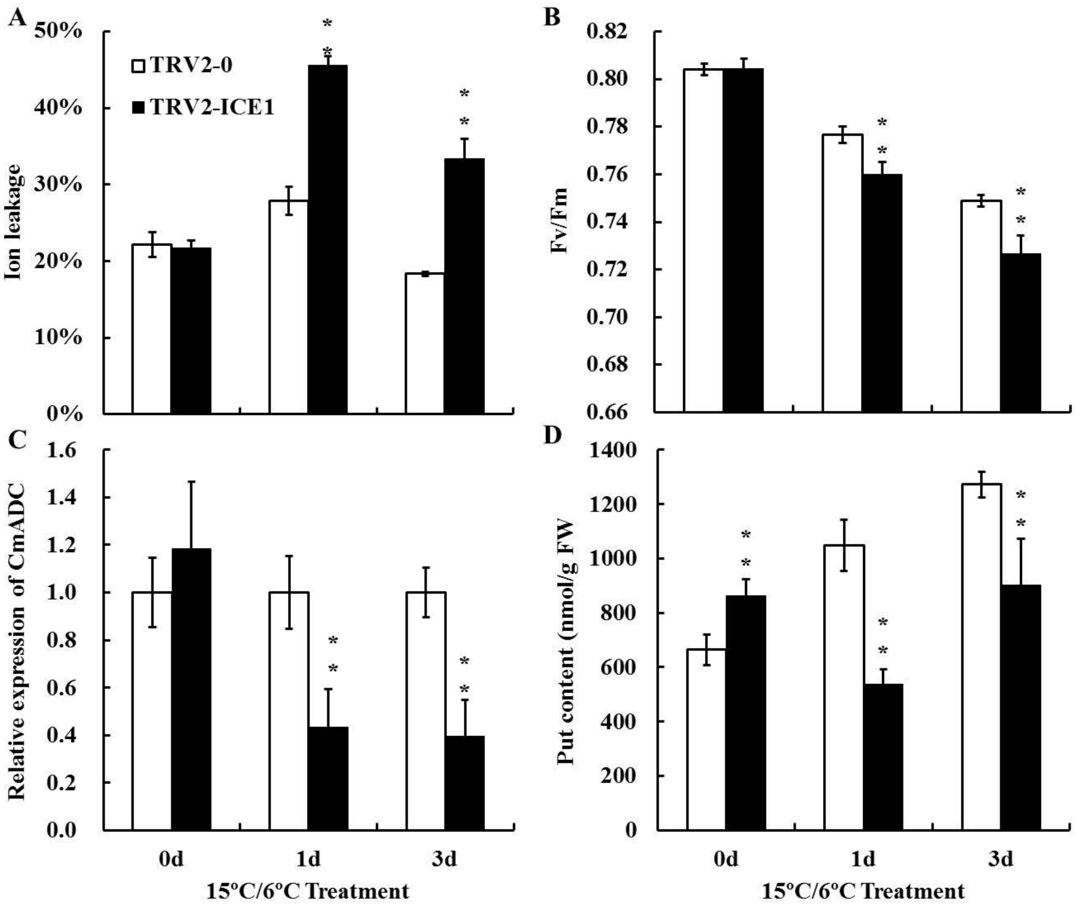

Fig.S6 VIGS of *CmICE1* resulted in the decrease of Put content and cold tolerance in melon seedlings. Ion leakage (**A**), Fv/Fm (**B**), *CmADC* expression(**C**) and Put accumulation (**D**) of melon seedlings under low temperature treatment (15°C/6°C). Independent t-test was used to analyze the difference between silenced plants and control plants (WT). Significant differences are marked with asterisks (***P* < 0.01; **P* < 0.05). Error bars are shown with the three biological replicates.
